# Influence of Mineral Fillers on the Curing Process and Thermal Degradation of Polyethylene Glycol Maleate–Acrylic Acid-Based Systems

**DOI:** 10.3390/polym17192675

**Published:** 2025-10-03

**Authors:** Gulsym Burkeyeva, Anna Kovaleva, Danagul Muslimova, David Havlicek, Abylaikhan Bolatbay, Yelena Minayeva, Aiman Omasheva, Elmira Zhakupbekova, Margarita Nurmaganbetova

**Affiliations:** 1 The Department of Organic Chemistry and Polymers, Chemistry Faculty, Karagandy University of The Name of Academician E.A. Buketov, Karaganda 100024, Kazakhstan; guls_b@mail.ru (G.B.); m.dana_777@mail.ru (D.M.); abylai_bolatbai@mail.ru (A.B.); yelenaminayeva@yandex.ru (Y.M.); valihanovna@mail.ru (A.O.); elmira_zhakupbek@mail.ru (E.Z.); ritunur@mail.ru (M.N.); 2 The Department of Inorganic Chemistry, Faculty of Science, Charles University, Albertov 2030, CZ-128 43 Prague 2, Czech Republic; david.havlicek@natur.cuni.cz

**Keywords:** unsaturated polyester resins, polyethylene glycol maleate, mineral fillers, thixotropic properties, curing kinetics, thermal stability

## Abstract

For the first time, the kinetics of isothermal curing and thermal degradation of polyethylene glycol maleate (pEGM)–based systems and their composites with mineral fillers were investigated in the presence of a benzoyl peroxide/N,N-Dimethylaniline redox-initiating system. DSC analysis revealed that the curing process at 20 °C can be described by the modified Kamal autocatalytic model; the critical degree of conversion (*α_c_*) decreases with increasing content of the unsaturated polyester pEGM and in the presence of fillers. In particular, for unfilled systems, *α_c_* was 0.77 for pEGM45 and 0.60 for pEGM60. TGA results demonstrated that higher pEGM content and the incorporation of fillers lead to increased thermal stability and residual mass, along with a reduction in the maximum decomposition rate (dTGₘₐₓ). Calculations using the Kissinger–Akahira–Sunose and Friedman methods also confirmed an increase in the activation energy of thermal degradation (E_a_): *E_KAS_* was 419 kJ/mol for pEGM45 and 470 kJ/mol for pEGM60, with the highest values observed for pEGM60 systems with fillers (496 kJ/mol for SiO_2_ and 514 kJ/mol for CaCO_3_). Rheological studies employing three-interval thixotropy tests revealed the onset of thixotropic behavior upon filler addition and an increase in structure recovery after deformation of up to 56%. These findings underscore the potential of pEGM-based systems for low-temperature curing and for the design of composite materials with improved thermal resistance.

## 1. Introduction

Modern requirements for polymer materials intended for structural and functional applications necessitate research into the synthesis and modification of high-performance thermosetting compositions with controllable rheological properties, strong adhesion, and resistance to aggressive environmental factors. Such materials are in high demand in construction, transportation engineering, as well as in the aerospace and shipbuilding industries [1,2,3,4]. Among thermosetting binders, particular attention is given to unsaturated polyester resins (UPRs), which, due to their relatively low viscosity, high reactivity, and ability to cure at moderate temperatures, form robust three-dimensional networks with excellent chemical and thermal resistance [5,6,7,8,9,10].

Most studies on UPRs have focused on systems cured with styrene or methyl methacrylate [11,12,13]. However, these monomers possess several significant drawbacks, including high volatility, toxicity, and substantial shrinkage during curing. Consequently, alternative approaches are being actively developed to reduce shrinkage deformations and improve the stability of polymer matrices. The introduction of low-shrinkage additives has proven effective in compensating for shrinkage [14]. The use of mineral fillers also makes it possible to control the viscosity, thixotropy, thermal conductivity, and thermal stability of polymer systems. Studies [15,16] have reported improvements in thixotropic properties through the incorporation of inorganic fillers. Other works [17,18] investigated the effect of mineral additives on UPRs and demonstrated the possibility of significantly modifying their rheological behavior. These findings highlight the importance and applied relevance of research aimed at developing advanced polymeric materials based on UPRs. However, despite the high potential of UPRs cured with styrene and methyl methacrylate [1,9,11,12], the number of studies involving other vinyl-containing monomers remains limited.

The use of unsaturated carboxylic acids in UPRs represents a promising research direction that requires further investigation. In particular, the copolymerization of unsaturated polyesters (UPs) with acrylic acid (AA) can significantly broaden the functionality of the resulting materials [7,10]. However, to date, no studies have addressed the influence of AA and mineral fillers on structural transformations, curing kinetics, or the rheological and thermal behavior of UPRs. The study of rheological properties is of great importance for evaluating processability, the ability of systems to recover their structure after mechanical deformation, and the optimization of processing conditions. To investigate the curing kinetics of thermosetting resins, Fourier-transform infrared spectroscopy (FTIR) [19,20,21], rheometry [22,23], and differential scanning calorimetry (DSC) [24,25,26,27] are commonly applied. Among these methods, DSC has become the most widely used tool for studying the entire curing process. During curing, the rate and degree of conversion as functions of time can be determined by approximating the exothermic effects recorded by DSC with different kinetic models [27]. The most widely applied models are the autocatalytic model proposed by Sourour and Kamal [24,27] and the modified autocatalytic model accounting for diffusion control, developed by Khanna et al. [28,29]. The literature provides some evidence on the thermal stability of UPR–AA copolymers [10,30]. However, no information is available regarding the thermal behavior of such systems with fillers in the presence of a redox-initiating system at room temperature. Data on curing kinetics and thermal stability are essential for determining processing parameters, optimizing heat-treatment conditions, and predicting the performance of the final materials under various temperature regimes.

Therefore, the aim of this study is to investigate the kinetic characteristics and thermal stability of thermosetting systems based on polyethylene glycol maleate and acrylic acid in the presence of inorganic fillers. Special attention is given to the effect of the nature and concentration of fillers (calcium carbonate and silicon dioxide) on the viscosity and thixotropic recovery of the studied systems.

## 2. Materials and Methods

### 2.1. Materials

Maleic anhydride (≥99%, Thermo Fisher Scientific, Waltham, MA, USA) and ethylene glycol (99.8%, Sigma-Aldrich, Burlington, VT, USA) were used as the starting monomers for the polycondensation reaction. Zinc chloride (≥98%, Sigma-Aldrich, Burlington, VT, USA) was employed as a catalyst in the polycondensation reaction. Acrylic acid (≥99%, Sigma-Aldrich, Burlington, VT, USA) was used as a vinyl-containing monomer for radical copolymerization with UP. Benzoyl peroxide Luperox^®^ A75 (Sigma-Aldrich, Burlington, VT, USA) was used to initiate the radical copolymerization. N,N-Dimethylaniline (99.5%, Thermo Fisher Scientific, USA) was used as a promoter to activate the curing process in the presence of the initiator.

Calcium carbonate (≥99%, Reaktivsnab, Shymkent, Kazakhstan) and silicon dioxide (Aerosil^®^ 300, Evonik Industries, Essen, Germany) were used as inorganic fillers.

### 2.2. Preparation of Polyethylene Glycol Maleate

**Figure 1:**
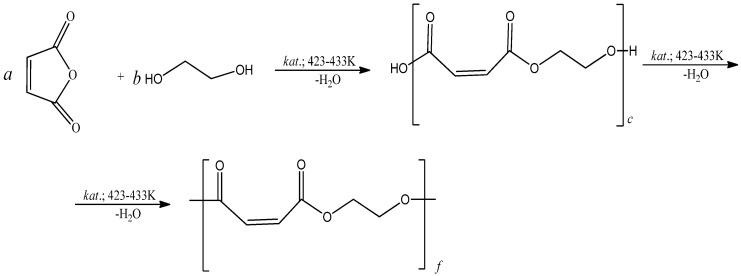
Scheme of polyethylene glycol maleate formation. Where c = a + b (the formation of acidic ester); f = a + b (the formation of polyester).

Polyethylene glycol maleate (pEGM) was obtained by polycondensation of ethylene glycol with maleic anhydride at 423–443 K. The polycondensation was carried out according to a standard procedure in the presence of zinc chloride as a catalyst under a nitrogen atmosphere [9]. The synthesis reaction and the most probable structure of the resulting polyester are shown in Figure 1.

### 2.3. Sample Preparation

An ultrasonic homogenizer (Scientz-IID, Ningbo Scientz Biotechnology Co., Ltd., Ningbo, China) was used for sample preparation: the required amounts of pEGM, acrylic acid (AA), the initiating system, and fillers were mixed at a frequency of 20–25 kHz for 30 min. The application of ultrasonic treatment ensured effective dispersion of the components and the formation of a homogeneous reaction mixture for subsequent studies and curing.

### 2.4. Curing of Polyethylene Glycol Maleate

Curing of the UP–AA reaction mixture was carried out by bulk radical copolymerization at various molar ratios of the comonomers, pEGM:AA: 45.3:54.7 wt.% (pEGM45) and 60.2:39.8 wt.% (pEGM60), at 20 °C in the presence of a redox initiating system consisting of benzoyl peroxide (BPO) and N,N-Dimethylaniline (DMA). The concentrations of the initiator (BPO) and the promoter (DMA) were 1% and 0.15%, respectively, relative to the total mass of the initial pEGM:AA mixture for all experiments. In addition, the filler concentrations were varied as follows: SiO_2_ at 5% and 10%, and CaCO_3_ at 15% and 30%.

### 2.5. Methods

#### 2.5.1. NMR Analysis

Nuclear magnetic resonance (NMR) spectra were recorded on a Bruker Avance III 600 MHz spectrometer (VTT) in deuterated chloroform (CDCl_3_). All measurements were performed at room temperature.

#### 2.5.2. Gel-Permeation Chromatography

The molecular weight of the synthesized pEGM was determined by gel permeation chromatography (GPC) using a Malvern chromatograph equipped with a Viscotek 270 dual detector (Malvern Instruments Inc., Bristol, UK), yielding an average molecular weight of approximately 1728 Da. Polystyrene was used as the calibration standard, and dioxane was employed as solvent [10].

#### 2.5.3. Rheological Analysis

The rheological properties of the pEGM-based systems were investigated using an MCR 302e rheometer (Anton Paar, Graz, Austria). Before the measurements, the samples were carefully mixed and allowed to rest for 5 min to release residual stresses induced during preparation. Each experiment was repeated at least three times to ensure reproducibility, and the deviation between parallel measurements did not exceed 5%. During the experiments, viscosity–shear rate curves were obtained, and a three-interval thixotropy test was performed in rotational mode to monitor structural recovery [31]. The measurements were carried out using a CP-40 measuring system (plate diameter 40 mm, gap height 0.08 mm). Structural recovery tests generally comprise three consecutive stages designed to determine the material’s ability to restore its structure after shear-induced breakdown. In the first stage, a low shear stress not exceeding the yield point was applied to the sample. In the second stage, a shear stress above the yield point was applied to break down the internal structure of the material. In the final stage, the stress was reduced to its initial low value, and structural recovery was monitored over a set period of time. Thixotropic measurements were performed in a controlled shear rate mode [32].

- Interval I (0–30 s): low shear rate (γ˙ = 0.1 s^−1^), allowing determination of the initial viscosity in the quiescent state;
- Interval II (30–60 s): high shear rate (γ˙ = 1000 s^−1^), simulating an external load that disrupts the material structure;
- Interval III (60–110 s): return to a low shear rate (γ˙ = 0.1 s^−1^), aimed at determining the material’s ability to restore its viscosity and, consequently, its internal structure.

All rheological tests were carried out at 20 °C, prior to the onset of curing, in the presence of the BPO/DMA redox initiating system. Temperature control of the system was maintained in a convection oven equipped with Peltier elements. The rheometer operation and data acquisition were performed using RheoCompass™ software 1.35.1394 (Anton Paar).

#### 2.5.4. Differential Scanning Calorimetry (DSC)

The thermal effects of the pEGM curing reaction were studied by DSC using a Labsys Evolution TG-DTA/DSC simultaneous thermal analysis system (Setaram Instrumentation, Caluire, France). A reaction mixture (95–100 mg) consisting of pEGM with AA, fillers, and the BPO/DMA redox initiating system was placed in open alumina crucibles and loaded into the calorimeter’s measuring cell. The tests were carried out under isothermal conditions at 20 °C in a nitrogen atmosphere. The curing reaction was considered complete when the exothermic curve returned to the baseline. The total area under the exothermic curve, calculated from the extrapolated baseline at the end of the reaction, was used to determine the isothermal heat of curing (${∆H}_{i}$) [33].

The degree of curing (α) is expressed as:

(1) $$\alpha =\frac{{∆H}_{i}}{{∆H}_{tot}},$$

where

(2) $$\Delta H_{tot}=\Delta H_{i}+\Delta H_{r}$$

$\Delta H_{r}$ is the residual reaction enthalpy obtained from dynamic scanning of the isothermally cured sample.

After completion of the isothermal experiment, the DSC cell was rapidly cooled to room temperature, and after stabilization, the residual heat of curing ($\Delta H_{r}$) was measured until no exothermic effect was observed. The $\Delta H_{r}$ value was recorded in dynamic mode at a heating rate of 10 °C/min over a temperature range from 20 °C to 200 °C.

The enthalpies were obtained by integrating the DSC exotherm and normalized to the mass of the reactive pEGM:AA mixture.

Each experiment was conducted three times. The kinetic parameters were calculated based on the resulting data and are presented as mean values with standard deviations, ensuring the reliability and reproducibility of the results.

The isothermal curing kinetics of the pEGM45 and pEGM60 systems at 20 °C were analyzed using the Kamal autocatalytic model according to Equation (3) [26,27]:

(3) $$\frac{d_{\alpha }}{d_{t}}=\left(k_{1}+k_{2}\alpha^{m}\right)\cdot (1-\alpha {)}^{n}$$

where

α is the degree of curing,

$k_{1}$ and $k_{2}$ are the apparent rate constants corresponding to the Arrhenius law,

m and n are the kinetic reaction orders.

In the present study, the parameters m, n, and $k_{2}$ were determined by fitting the experimental data using a graphical method and nonlinear curve fitting. The constant $k_{1}$ can be calculated from the initial reaction rate at α = 0.

Equation (3) can be rewritten as:

(4) $$ln\left(\frac{d_{\alpha }}{d_{t}}\right)=ln\left(k_{1}+k_{2}\alpha^{m}\right)+nln\left(1-\alpha \right)$$

The value of *n* can be obtained from the slope of the plot of $ln\left(\frac{d_{\alpha }}{d_{t}}\right)$ versus $ln\left(1-\alpha \right)$.

Equation (3) can also be expressed as:

(5) $$ln\left\{\left[\left(\frac{d_{\alpha }}{d_{t}}\right)/(1-\alpha {)}^{n}\right]-k_{1}\right\}=\ln k_{2}+m\ln\alpha$$

Plotting the left-hand side of Equation (5) as a function of lnα yields a straight line, from whose slope and intercept the values of m and $k_{2}$ can be determined, respectively.

#### 2.5.5. IR-Spectroscopy

FTIR (Fourier Transform Infrared Spectroscopy) spectra of the samples were recorded on a Thermo Fisher Scientific Nicolet iS50 FTIR spectrometer (Waltham, MA, USA) (4 cm^−1^ resolution, Happ–Genzel apodization) in the ranges 400–4000 cm^−1^ (KBr beamsplitter) and 100–1800 cm^−1^ (Solid Substrate™,Thermo Fisher Scientific, Waltham, MA, USA). Measurements were carried out using the ATR technique with a diamond crystal, and standard ATR correction was applied to the recorded spectra [34].

#### 2.5.6. Thermogravimetric Analysis (TGA)

The thermal properties of the systems under study were investigated using a Labsys Evolution TG-DTA/DSC simultaneous thermal analysis instrument (Setaram Instrumentation, Caluire, France) in dynamic mode over a temperature range of 20–600 °C. The samples were heated in Al_2_O_3_ crucibles at heating rates of 5, 10, and 20 °C/min in a nitrogen atmosphere with a flow rate of 30 mL/min. Calibration of the instrument for thermogravimetric measurements and heat flow was carried out using CaCO_3_ and indium (In) standards, respectively. Each measurement was performed in triplicate.

The kinetics of thermal decomposition are generally expressed by the following equation [35,36]:

(6) $$\frac{d\alpha }{dt}={Aexp}^{-\frac{E_{a}}{RT}}\cdot f(\alpha ),$$

The resulting equation provides the basis for differential kinetic methods. The right-hand side of Equation (6) cannot be solved analytically; therefore, various approximation methods are used in practice. The Friedman differential method was derived using an isoconversional approach to Equation (6), the results of which are presented in [37,38]:

(7) $$In{\left(\frac{da}{dt}\right)}_{\alpha ,\;i}\;=\;In\left[f\left(\alpha \right)A_{\alpha }\right]\;-\;\frac{E_{a}}{{RT}_{a,\;i}}$$

The subscript *i* is introduced to denote different temperature programs. *T_a,i_* is the temperature at which the conversion (*α*) is reached for temperature program *i*. As a result, Equation (7) assumes that *T_a,i_* changes linearly over time at a heating rate *β_i_*.

There are several integral isoconversional methods that differ in the approximation of the temperature integral in Equation (7). The Murray and White approximation yields B = 2 and C = 1, which leads to another well-known equation, often referred to as the Kissinger–Akahira–Sunose equation [37,38,39,40]:

(8) $$ln\left(\frac{\beta_{i}}{T_{a,i}^{2}}\right)\;=\;Const\;-\;\frac{E_{a}}{{RT}_{a,i}}$$

The activation energy for different degrees of conversion is calculated from the slope of the plot of $ln\left(\frac{\beta_{i}}{T_{a,i}^{2}}\right)$ versus 1/*T*.

## 3. Results and Discussion

### 3.1. Curing of pEGM: AA Systems

**Figure 2:**
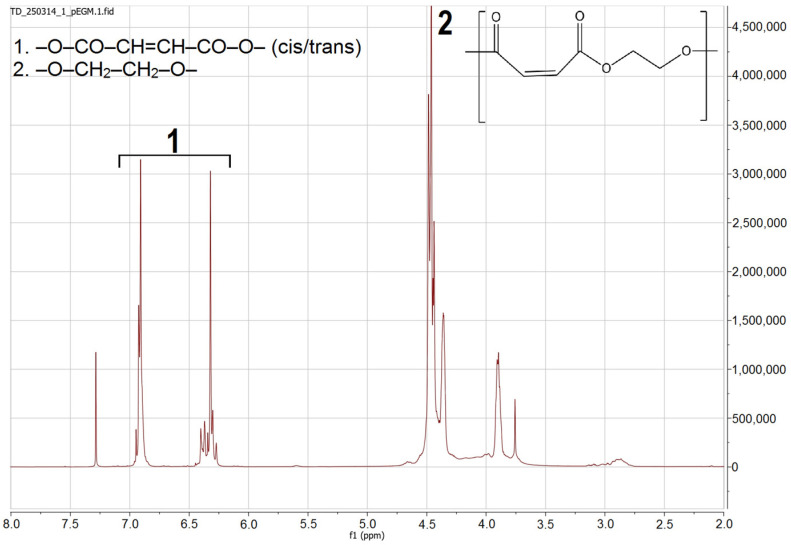
^1^H NMR spectrum of pEGM.

^1^H and ^13^C NMR spectroscopy was used to characterize the synthesized pEGM. Analysis of the ^1^H NMR spectrum (Figure 2) revealed signals corresponding to protons of various functional fragments within the polymer structure. Characteristic chemical shifts were identified, confirming the presence of vinyl, methylene, and carbonyl groups. In the interpretation and quantitative evaluation of the oligomer structure, the presence of several types of protons was taken into account.

In the region of δ 6.20–6.40 ppm, a signal corresponding to the vinylic protons of the maleate units –O–CO–CH=CH–CO–O– is observed, appearing as an almost singlet due to symmetry. The vinylic protons of the fumarate units (trans-isomer formed through thermal isomerization of maleate fragments) are detected at δ 6.70–7.00 ppm. The main broad signal at δ 4.30–4.70 ppm corresponds to the methylene protons –O–CH_2_–CH_2_–O– adjacent to the ester groups, which represent the polyester matrix.

**Figure 3:**
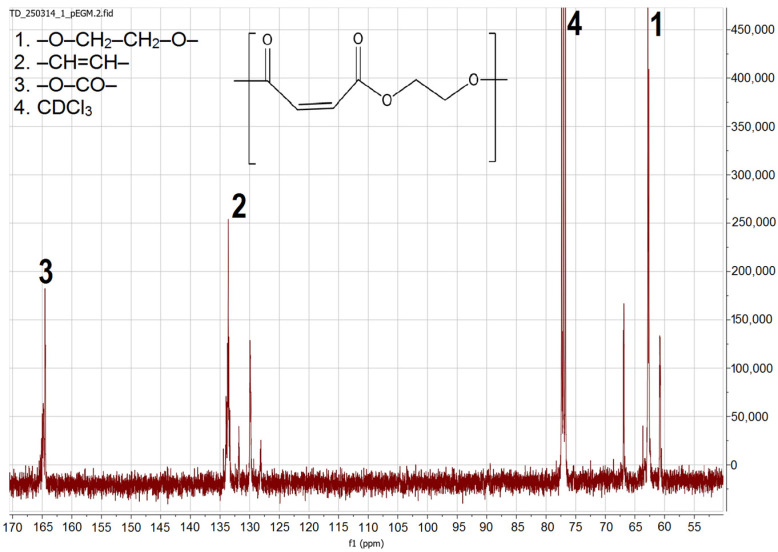
^13^C NMR spectrum of pEGM.

The ^13^C NMR spectrum (Figure 3) displays three groups of resonance signals corresponding to different types of carbon atoms in the pEGM structure. Signals in the δ 164–169 ppm region are attributed to the carbonyl carbons of the ester groups(–O–CO–) in maleate/fumarate units, with two closely spaced resonances arising from cis/trans environments. The vinylic carbons (–CH=CH–) appear at δ 127–137 ppm, confirming the presence of unsaturated segments within the polymer structure. Signals in the δ 62–66 ppm range correspond to methylene carbons (–O–CH_2_–CH_2_–O–) of the ethylene glycol chain. The intense solvent peak of CDCl_3_ is observed at δ 77.0 ppm. The overall set of signals verifies the structure of the unsaturated polyester based on maleate/fumarate units and indicates the absence of residual anhydride.

**Figure 4:**
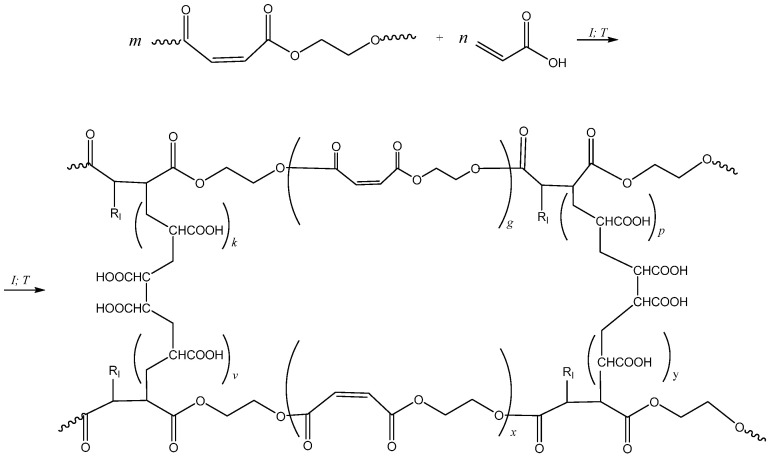
Synthesis scheme of the pEGM–AA system. R_I_ denotes the initiator radical.

Figure 4 presents the scheme of the radical copolymerization reaction of pEGM with AA. Curing of UPRs based on pEGM is carried out at 20 °C in the presence of the BPO/DMA redox initiating system.

### 3.2. Results of Rheological Analysis 

The rheological properties of binder materials determine many performance characteristics of composite materials, such as paints and coatings, sealants, adhesives, and others [2]. In the quiescent state, these materials should have sufficiently high viscosity to prevent spontaneous spreading. However, under external mechanical action, their viscosity should decrease, ensuring ease of processing and application. Another important factor is the ability of the material to rapidly restore its initial viscosity after the load is removed. In this context, the rheological characteristics of the systems under study were investigated. In particular, the dependence of viscosity on shear rate and the thixotropic properties were examined. Viscosity recovery tests were carried out in a three-interval mode, comprising shear-induced structural breakdown, subsequent recovery, and final stabilization.

**Figure 5:**
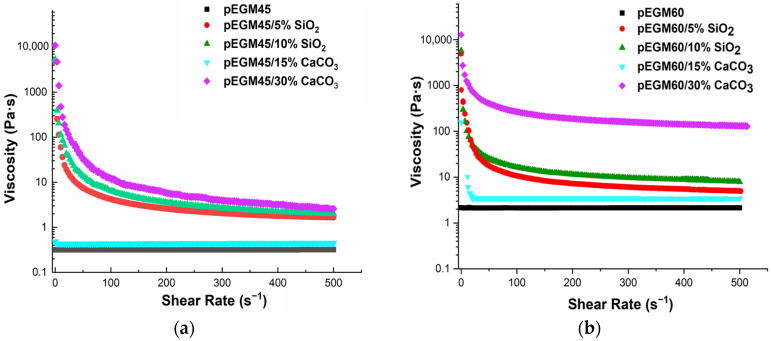
Viscosity–shear rate dependence for the systems: (**a**) pEGM45; (**b**) pEGM60.

Figure 5 shows the plots of apparent viscosity versus shear rate for the pEGM45 and pEGM60 systems and their compositions with fillers.

Silicon dioxide and calcium carbonate were used as fillers, with concentrations chosen according to their nature and activity. Silicon dioxide is a lightweight, finely dispersed filler with a high specific surface area. It exhibits a pronounced structuring effect even at 5% (the lower boundary of the percolation region) [41]. The upper boundary of the percolation range for silicon dioxide is approximately 10%, corresponding to the so-called “saturation concentration”. In contrast, macrodisperse and inert calcium carbonate exhibit a noticeable effect only at substantially higher concentrations. Thus, in [42] it was reported that the mechanical properties of the composite vary at CaCO_3_ concentrations ranging from 10 to 50%, with substantial modifications already observed at levels up to 40%. Similarly, in a study on CaCO_3_-filled composites [43] filler loadings of up to 35% were employed. These findings support the methodological rationale for selecting such ranges of filler contents.

The obtained rheograms show that the pEGM45 and pEGM60 systems without fillers exhibit Newtonian behavior, with a constant viscosity value over the investigated shear rate range, indicating the absence of structural changes in the flow. This is attributed to the relatively low molecular weight of the polyester synthesized by us [44]. The comparatively short polyester chains do not provide sufficient interchain entanglement [45], which limits the structuring of the system. This behavior is consistent with Newton’s law of viscosity [46] (*τ* = *η* · *ẇ*), where *η* is the dynamic viscosity.

The incorporation of silicon dioxide into the pEGM45 and pEGM60 systems results in the appearance of pseudoplastic behavior, in which viscosity decreases with increasing shear rate. This effect is more pronounced at an SiO_2_ concentration of 10% than at 5%. The pronounced decrease in viscosity with increasing shear rate confirms that SiO_2_ forms a three-dimensional network [47] that breaks down under shear. This behavior is described by the Ostwald–de Waele power-law model ($\tau =k\cdot \dot{\gamma^{n}}$, *n* < 1).

Calcium carbonate at concentrations of 15% and 30% has different effects on the rheological characteristics of the systems. It is noteworthy that pEGM45/15% CaCO_3_ retains Newtonian behavior with constant viscosity, indicating the absence of significant interparticle interactions in the system. The pEGM60/15% CaCO_3_ system exhibits behavior close to that of Newtonian liquids: at low shear rates, a slight decrease in viscosity is observed, followed by a constant viscosity over the entire shear rate range. This indicates a low degree of structural organization, which may be attributed both to the relatively low filler concentration and to the absence of specific interactions between calcium carbonate and the polymer matrix. This effect may be associated with the breakdown of filler aggregates formed in the system under static conditions. Under shear, these aggregates are disrupted, leading to a reduction in the degree of structuring and a decrease in viscosity [48]. At this filler content, the aggregates do not form a stable three-dimensional network capable of responding to shear deformation. As a result, the flow remains uniform and independent of the applied shear rate.

The pEGM45/30% CaCO_3_ and pEGM60/30% CaCO_3_ systems exhibit pseudoplastic behavior, with viscosity decreasing as the shear rate increases. A reduction in viscosity is observed across the entire shear rate range as deformation increases. This may be attributed to the formation of a partial structural network resulting from dense packing and physical contact between filler particles at high calcium carbonate content. The low AA content in pEGM60 reduces the likelihood of forming intermolecular interactions between the matrix and the filler, which also facilitates shear-induced breakdown. Taken together, these factors lead to reduced flow resistance and the manifestation of pseudoplastic liquid behavior.

Thus, the rheological properties of the modified compositions under study depend significantly on both the composition of the pEGM systems and the nature and concentration of the filler. Silicon dioxide has a high specific surface area and the ability to engage in interparticle interactions through the formation of hydrogen bonds. The introduction of this filler promotes the formation of a three-dimensional network structure that is sensitive to shear deformation. This results in pseudoplastic behavior, which becomes more pronounced with increasing SiO_2_ concentration.

Calcium carbonate does not significantly affect the rheological properties of pEGM systems. At a CaCO_3_ concentration of 15%, its interaction with the pEGM polymer matrix is minimal, resulting in the retention of Newtonian behavior in the solution. At a CaCO_3_ concentration of 30%, a weak structure is formed due to hydrogen bonding between CaCO_3_ particles and AA, leading to pseudoplastic behavior. However, as the AA content decreases, the number of possible interactions is reduced, which manifests as a weakly pronounced pseudoplastic effect in the pEGM60/30% CaCO_3_ system.

The obtained data show that variation in composition, primarily in the content of functional groups capable of interacting with the filler, makes it possible to control the rheological properties of the system, which is important for producing materials with the desired characteristics.

**Figure 6:**
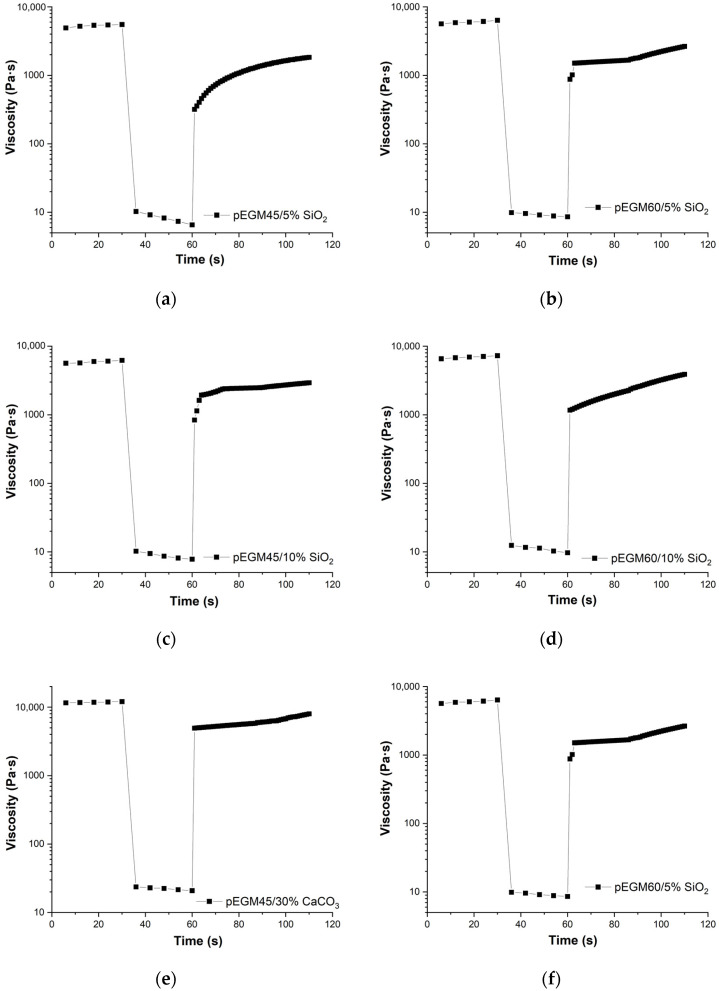
Rheograms from the three-interval thixotropy test for pEGM-systems: (**a**) pEGM45/5% SiO_2_, (**b**) pEGM60/5% SiO_2_, (**c**) pEGM45/10% SiO_2_, (**d**) pEGM60/10% SiO_2_, (**e**) pEGM45/30% CaCO_3_, (**f**) pEGM60/5% CaCO_3_.

Figure 6 presents the results of the three-interval thixotropy test. The studies were carried out for systems that exhibited pseudoplastic behavior.

Three-interval thixotropic tests in rotational mode made it possible to quantitatively assess the dynamics of shear-induced breakdown and recovery in the pEGM polymer compositions. Figure 6 shows the viscosity–time curves for the pEGM45 and pEGM60 systems containing 5% and 10% SiO_2_, as well as 30% CaCO_3_. The viscosity changes over time display three distinct stages characteristic of thixotropic behavior: high initial viscosity, a sharp decrease at high shear rates, and partial recovery after returning to low deformation.

The results (Figure 6) showed that all studied formulations exhibited thixotropic properties, characterized by varying degrees of viscosity recovery after high-shear deformation.

The pEGM45/5% SiO_2_ and pEGM60/5% SiO_2_ systems exhibited structural recovery values of 25% and 28%, respectively. For the pEGM45 and pEGM60 systems with an SiO_2_ concentration of 10%, the recovery values were higher, at 35% and 40%, respectively. The presence of thixotropic properties is attributed to the formation of hydrogen bonds between SiO_2_ particles and the carboxyl groups of AA. In addition, SiO_2_ particles can form bonds with each other. Such interactions may lead to the formation of a temporary three-dimensional network structure that is disrupted under shear and re-forms in the quiescent state [49]. Thus, as the silicon dioxide content increases, the degree of structural recovery also increases.

The solutions containing calcium carbonate demonstrated the highest structural recovery values. The pEGM45/30% CaCO_3_ and pEGM60/30% CaCO_3_ samples had recovery values of 50% and 56%, respectively. This is explained by the fact that the high viscosity of the calcium carbonate-containing systems stabilizes the dispersed structure and reduces particle mobility, thereby promoting more effective structural recovery [34]. It should be noted that these results represent the degree of structural recovery 30 s after the start of the third interval; complete or more pronounced recovery may occur over a longer period.

### 3.3. Isothermal and Dynamic DSC Results

The kinetic parameters of the curing reaction for the pEGM45 and pEGM60 systems and their compositions with fillers were studied by DSC [50,51]. During the curing of thermosetting polymers, several processes may be observed, including the induction period, gelation, vitrification, thermal degradation, and others [50,52].

**Figure 7:**
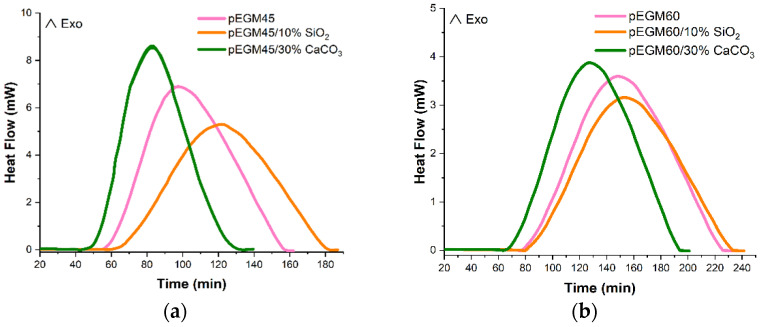
Isothermal DSC thermograms of the curing process for (**a**) pEGM45; (**b**) pEGM60.

Figure 7 shows the isothermal curing thermograms of the pEGM samples at 20 °C. Analysis of the DSC results indicates that the composition of the formulation has a significant effect on the curing reaction kinetics. At the initial stage, after the introduction of the BPO/DMA redox initiating system into the reaction mixture, an induction period is observed during which polymerization is negligible. During this period, the properties of the composition change only slightly. This is followed by the active phase of copolymerization, the rate of which, as well as the duration of the induction period, is determined by the rate of free radical formation, the temperature of the medium, and the monomer ratio in the initial mixture.

Table 1 presents the thermal characteristics of the curing process for pEGM-based systems with varying AA content and inorganic fillers. According to the DSC results, the pEGM45 systems are characterized by higher degrees of conversion compared to pEGM60. The pEGM45 systems exhibit higher reactivity, indicating a more complete curing reaction at 20 °C.

The thermal effects of the curing reaction depend on the pEGM:AA ratio and the type of filler. The pEGM45 systems are characterized by a higher total heat effect (∆*H_tot_*) than the pEGM60 formulations. In addition, the degree of curing (*α*) for pEGM45 is 0.84, which is significantly higher than that for pEGM60 (0.67).

As noted above, the introduction of fillers affects the thermal characteristics. The presence of silicon dioxide and calcium carbonate fillers reduces the total heat release (∆*H_tot_*). This reduction in ∆*H_tot_* may be attributed to a decrease in the concentration of reactive groups as a result of filler incorporation. The addition of silicon dioxide to the pEGM45 system reduces the total heat of reaction to 273.17 J/g, while the addition of calcium carbonate decreases it to 280.21 J/g. However, the degree of curing remains almost unchanged, at 0.83 and 0.82, respectively. A similar pattern is observed for the pEGM60 system: silicon dioxide lowers ∆*H_tot_* to 266.59 J/g, and calcium carbonate to 272.21 J/g, while the degree of curing remains at 0.66 and 0.65, respectively. These data indicate that the pEGM45-based systems are more thermoreactive compared to the pEGM60 systems. It should be noted that the nature of the fillers influences the curing time. As evidenced by the thermograms (Figure 7), silica slightly delays the curing process of pEGM compositions compared with the unfilled systems. This retardation is caused by adsorption of the initiator or accelerator on the filler surface, which reduces the concentration of active components in their free form and hinders the initiation of radical polymerization. In addition, radical inhibition occurs due to interactions between free radicals and the silicon dioxide surface.

Calcium carbonate slightly accelerates the curing process (Figure 7). One possible reason is the pH of the medium at the filler surface: calcium carbonate exhibits mildly basic surface properties [48], which promote faster decomposition of the initiator in the presence of the promoter. As a result, active species (radicals) that initiate polymerization are generated more rapidly, leading to a more active curing process.

**Figure 8:**
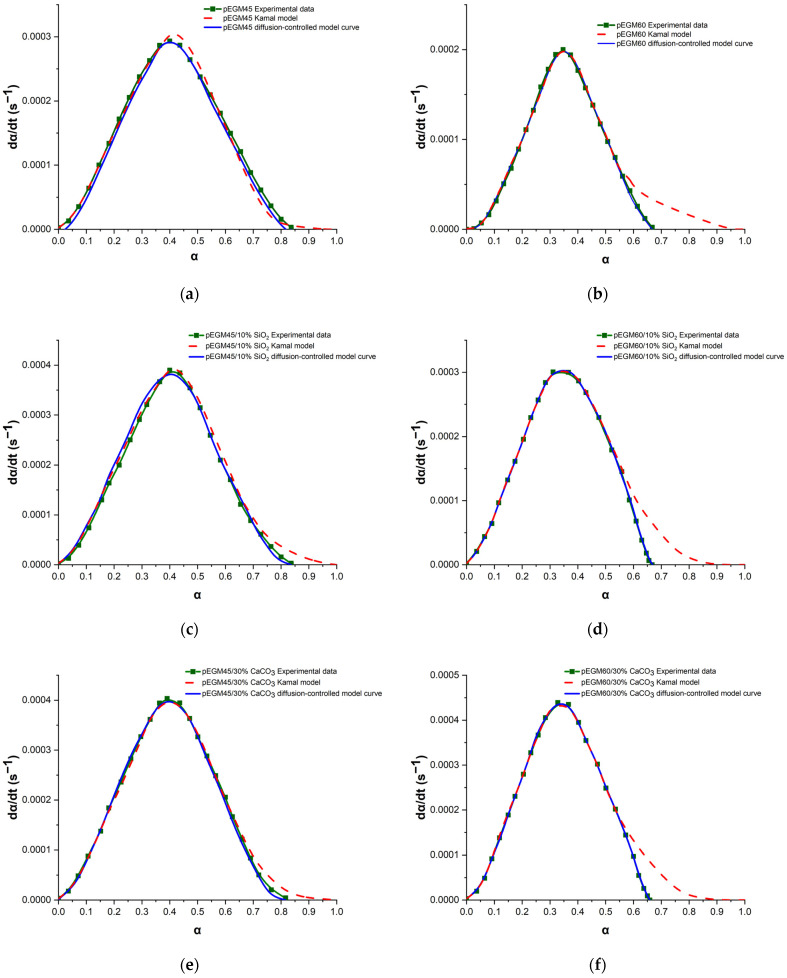
Plots of dα/dt versus α for the pEGM-systems: (**a**) pEGM45, (**b**) pEGM60, (**c**) pEGM45/10% SiO_2_, (**d**) pEGM60/10% SiO_2_, (**e**) pEGM45/30% CaCO_3_, (**f**) pEGM60/30% CaCO_3_.

The Kamal autocatalytic model was used to describe the isothermal curing process and evaluate the kinetic parameters of the pEGM systems. Table 2 presents the values of the parameters *k*_1_, *k*_2_, *m* and *n*, calculated using the above-described method for the pEGM45 and pEGM60 systems. As can be seen, these parameters depend on the composition of the reaction mixtures. To assess the validity of the model, the experimental data were compared with theoretical curves calculated using the Kamal autocatalytic model. The curves obtained with the autocatalytic model and the parameters from Table 2 were plotted for comparison with the experimental results (Figure 8). At the initial curing stages, when chemical control predominates, the autocatalytic model shows good agreement with the experimental data. However, at later stages of the process, deviations are observed, indicating a transition to a diffusion-controlled regime. As the curing reaction of the studied systems proceeds, gelation and vitrification occur due to an increase in viscosity, crosslink density, and the molecular weight of the UPR. The post-gel stage is associated with the possible onset of diffusion control in the curing kinetics of UPRs [53,54,55].

To account for the effect of diffusion control at the later stages of curing, a diffusion factor *f*(*α*) was introduced into the Kamal autocatalytic model [56,57]:

(9) $$\frac{d_{\alpha }}{d_{t}}=\left(k_{1}+k_{2}\alpha^{m}\right){\left(1-\alpha \right)}^{n}\cdot f\left(\alpha \right)$$

where *f*(*α*) is given by:

(10) $$f\left(\alpha \right)=\frac{1}{1+\exp\left[C\left(\alpha -\alpha_{C}\right)\right]}$$

where

*C* is the modification constant,

*α_c_* is the critical degree of curing determined by the glass transition temperature (*T_g_*) of the system. The value of *f*(*α*) can be obtained by dividing the experimental data by the values calculated using the Kamal model. The parameters *C* and $\alpha_{c}$ are determined by fitting the data according to Equation (10).

Figure 8 shows the dependence of the conversion rate (*d_α_*/*d_t_*) on the degree of curing (*α*) for the pEGM45 (a) and pEGM60 (b) systems obtained at 20 °C. The experimental data (square markers) are compared with theoretical curves calculated using the Kamal autocatalytic model (dashed red line) and the modified model accounting for diffusion limitations (solid blue line) in accordance with Equation (9).

For the pEGM45 system (Figure 8a), the maximum degree of curing reaches α_max_ ≈ 0.84, with the Kamal model showing good agreement with the experimental data in the initial and intermediate conversion regions. However, at the final stage (*α_c_* ≈ 0.77), a slight deviation is observed: the autocatalytic model overestimates the conversion rate, whereas the modified model incorporating the diffusion factor *f*(*α*) more accurately reflects the observed slowdown. This indicates the onset of diffusion limitations at the later stage of curing.

For the pEGM60 system (Figure 8b), the degree of conversion is lower (*α*_max_≈ 0.67), and the influence of diffusion is significantly more pronounced. The Kamal model substantially overestimates the conversion rate at *α_c_* ≈ 0.60, whereas the modified model accurately reproduces the shape of the experimental curve across the entire range. This indicates pronounced vitrification or an increase in viscosity, which limits the mobility of the reactants and leads to diffusion-controlled curing.

Thus, in both systems, diffusion-induced slowdown is observed at the later curing stages, requiring the inclusion of an appropriate correction factor in the model. The application of the modified model with *f*(*α*) makes it possible to adequately describe the curing kinetics under both moderate and pronounced diffusion limitations.

It should be noted that the kinetic behavior of the systems containing fillers (SiO_2_ and CaCO_3_) also conforms to the modified autocatalytic model that accounts for diffusion limitations (Figure 8c–f). The presence of fillers leads to a decrease in the critical degree of conversion (α_c_) due to several factors. First, the filler particles act as physical barriers, hindering the free diffusion of monomers and radicals within the reaction medium, which results in local restrictions on the mobility of polymer chains. Second, filler surfaces may adsorb initiators, monomers, and low-molecular-weight reaction products, thereby reducing the effective concentration of reactive groups in the resin volume. Third, the presence of a solid dispersed phase increases the effective viscosity of the system, accelerating the onset of diffusion-controlled behavior during the post-gel stage [58].

For systems containing silicon dioxide (Figure 8c,d), which possesses a high specific surface area and the ability to form hydrogen bonds with polar groups, the retardation of curing is more pronounced due to specific interactions between the filler particles and the functional groups of the polymer matrix [59]. In the case of calcium carbonate (Figure 8e,f), the determining factor is its high loading level (15–30%), which results in a pronounced increase in viscosity and a reduction in the free volume available for the reaction [60]. Thus, the presence of fillers shifts the critical degree of conversion to lower values and enhances the manifestation of diffusion control, which must be taken into account when describing the kinetics and modeling the curing behavior of composite materials.

**Figure 9:**
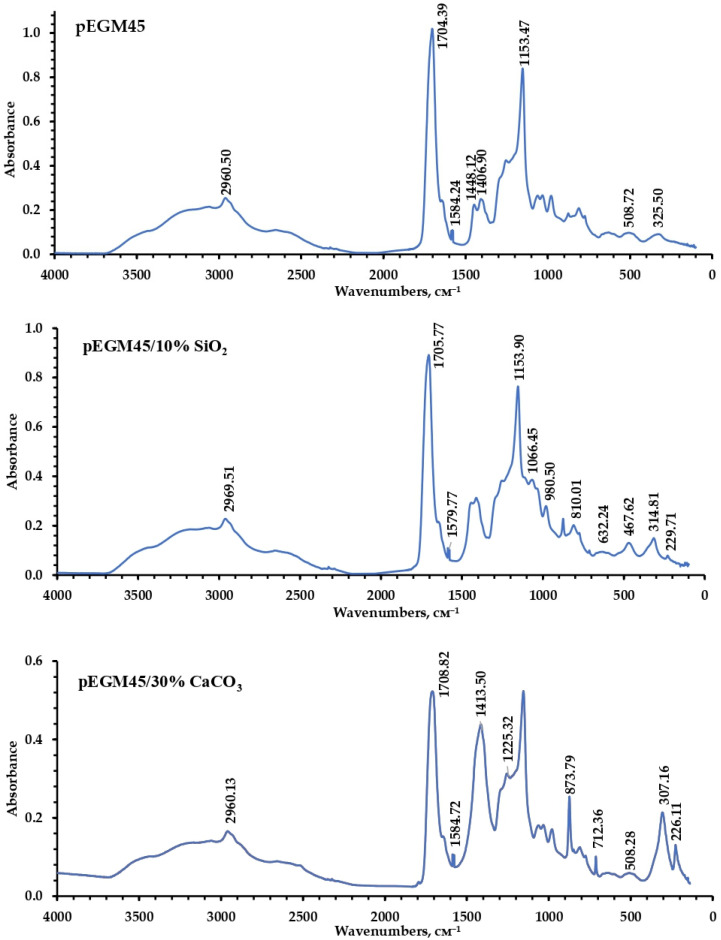
IR spectra of pEGM45, pEGM45/10% SiO_2_, and pEGM45/30% CaCO_3_.

To characterize the chemical structure of the synthesized polymer systems and their compositions, IR spectroscopy was performed in the mid-frequency range. Figure 9 presents the IR spectra of the pEGM45 sample without filler and the pEGM45/10% SiO_2_ and pEGM45/30% CaCO_3_ compositions. The analysis of the spectra reveals the presence of chemical groups characteristic of both the polymer matrix and the incorporated fillers.

In the IR spectra of the cured samples, characteristic absorption bands reflecting their structural features are observed. The bands at 1586–1589 cm^−1^ correspond to a certain amount of unreacted unsaturated double bonds of pEGM. Narrow signals in the 2960–2961 cm^−1^ region are assigned to the asymmetric and symmetric stretching vibrations of methylene groups (–CH_2_–), indicating the presence of aliphatic units in the macromolecules. A broad, intense band in the range of 2500–3300 cm^−1^ is attributed to the stretching vibrations of O–H groups of AA carboxyl functionalities and partially overlaps with the stretching vibrations of the methylene groups. In addition, in the IR spectra of all studied samples, the absorption region at 1704–1708 cm^−1^, corresponding to the carbonyl fragment (C=O) of the AA carboxyl group (–COOH), appears broadened and partially overlaps with the carbonyl band of the polyester matrix, which typically absorbs in the higher-frequency region (1730–1750 cm^−1^). In the 1100–1155 cm^−1^ range, C–O–C stretching vibrations are observed, indicating the presence of polyester fragments that form the structural backbone of the copolymer [34]. The combined spectral data confirm the presence of aliphatic, polyester, and carboxyl fragments in the structure of the obtained samples. The presence of these bands is observed in all samples, indicating the preservation of the primary structural organization of the polymer matrix after the incorporation of fillers.

The IR spectrum of the pEGM45/10% SiO_2_ sample is characterized by bands at 1066, 988, 810, and 632 cm^−1^, corresponding to the vibrations of Si–O–Si bonds.

In the spectrum of the pEGM45/30% CaCO_3_ composition, characteristic bands are observed at 1413, 873, and 712 cm^−1^, corresponding to the stretching and bending vibrations of the carbonate ion (CO_3_^2−^). Additional signals in the low-frequency region (500–600 cm^−1^) are attributed to Ca–O bond vibrations, typical of carbonate forms of calcium.

Analysis of the IR spectra made it possible to reliably identify the functional groups [61,62] characteristic of the polymer matrix. The characteristic peaks in the IR spectra also confirmed the presence of the inorganic components introduced into the system.

### 3.4. Results of TGA

To investigate the thermal properties of the synthesized pEGM-based systems, thermogravimetric analysis of the cured samples was carried out under linear heating conditions. The obtained data made it possible to assess the thermal stability of the studied systems and the nature of their degradation.

**Figure 10:**
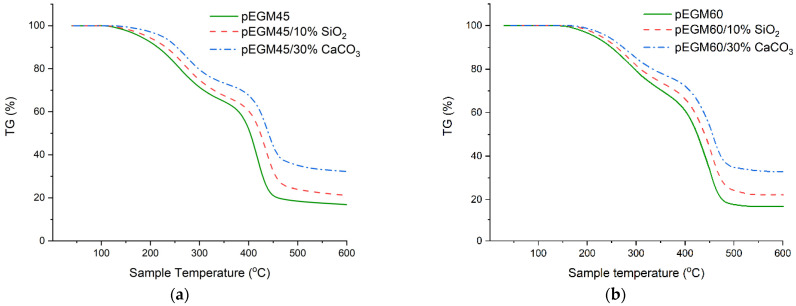
TG curves of the systems at a heating rate of 20 °C/min in an inert atmosphere: (**a**) pEGM45; (**b**) pEGM60.

As shown in Figure 10, the highest residual mass is observed for samples containing inorganic fillers, which is attributed to their high inertness as well as the high decomposition temperatures of CaCO_3_ and SiO_2_. The residual masses of the unfilled pEGM45 and pEGM60 samples were 15.7% and 16.5%, respectively, which is significantly lower than those of the filled systems. Specifically, the residual masses of the systems filled with silicon dioxide were 21.2% and 23.2%, while the pEGM45 and pEGM60 systems filled with calcium carbonate exhibited the highest residual masses of 32.3% and 33.6%, respectively.

**Figure 11:**
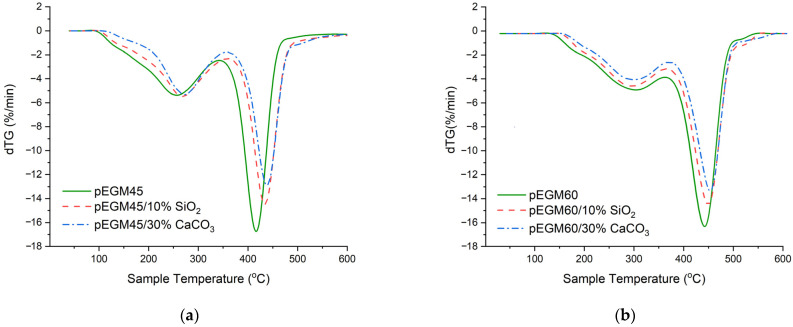
dTG curves of the systems at a heating rate of 20 °C/min in an inert atmosphere: (**a**) pEGM45; (**b**) pEGM60.

Figure 11 shows the dTG curves of the samples, providing a detailed representation of the decomposition stages.

As seen in Figure 11, the pEGM45 and pEGM60 polymer systems exhibit differences in thermal stability, which are reflected both in the temperature ranges of degradation and in the decomposition rates. For pEGM45, the first stage of thermal degradation begins at 90°C and ends at about 350°C, while the second stage occurs in the range of 350~475°C. The maximum decomposition rate is 16.7%/min at approximately 420 °C. In the case of pEGM60, degradation starts later: the first stage takes place in the range of 130~380 °C, and the second in the range of 380~515 °C (dTG_max_ = 16.4 %/min). Thus, the pEGM60 system exhibits higher thermal stability, as evidenced by the higher onset and completion temperatures of degradation at a comparable decomposition rate.

The addition of silicon dioxide has a stabilizing effect on both systems; however, the extent of this effect varies depending on the matrix composition. In the pEGM45/10% SiO_2_ system, the onset of degradation shifts to approximately 95 °C, while the second stage begins at around 365 °C with a mass loss of about 35%. The decomposition rate decreases to 14.5%/min. In the pEGM60/10% SiO_2_ system, degradation starts at 135 °C, and the second stage occurs in the range of 390~525 °C. The presence of silicon dioxide in the polymer matrix leads to a reduction in dTG_max_ and an increase in residual mass in both systems, indicating partial suppression of thermal degradation.

The introduction of calcium carbonate has the most pronounced effect on thermal stability, significantly reducing the decomposition rate and increasing the residual mass. In the pEGM45/30% CaCO_3_ system, the first stage begins at 105 °C and the second at 360 °C. In the pEGM60/30% CaCO_3_ system, the process starts at 140 °C and ends at 560 °C. The decomposition rates of the two stages are 4.2 %/min and 13.4 %/min at 300 °C and 450 °C, respectively. The substantial increase in residual mass, more than twice that of the unfilled systems, indicates a high proportion of non-decomposable components and the effective stabilizing action of the filler. Table 3 presents comparative thermogravimetric analysis data for the studied systems.

Based on the provided data, it can be noted that the thermal degradation of pEGM-based systems involves the following chemical processes: removal of moisture and low-molecular-weight volatile impurities, as well as the initial breakdown of weak bonds in the polymer structure during the first stage. In the second stage, bond cleavage occurs in the main macromolecular backbone—C–C, C–O, and other heteroatom bonds [63]. These processes are accompanied by the formation of volatile products, including olefin-type fragments, aldehydes, ketones, CO, and CO_2_. The degradation proceeds via a radical mechanism involving elements of depolymerization and rearrangements [64].

The presence of inorganic fillers has a noticeable effect on the thermal stability of the polymer matrix. Compared to the unfilled polymer matrix, the incorporation of amorphous silica contributes to a partial increase in thermal stability due to the adsorption of moisture and volatile degradation products on the particle surface. This process slows down their release into the gas phase and reduces the decomposition rate. In addition, the low thermal conductivity of silica limits heat transfer within the sample, leading to localized overheating, which is manifested as broadened transitions on the TG/dTG curves. As a result, SiO_2_-filled composites exhibit an increased residual mass and a slight shift in the degradation stages, although the onset and completion temperatures of the processes remain approximate. Silicon dioxide does not participate in chemical reactions; however, it acts as a thermal barrier, limiting macromolecular mobility and suppressing the propagation of thermodestructive radicals [65].

Calcium carbonate is also chemically inert within the studied temperature range and serves a stabilizing role, promoting the formation of a thermally stable residue [66]. As a result of its incorporation, a substantial increase in residual mass and a reduction in the thermal degradation rate are observed. Compared to the unfilled matrix, the addition of CaCO_3_ also enhances the thermal stability of the system. The high thermal conductivity of calcium carbonate promotes uniform heat distribution, which reduces the likelihood of local overheating and ensures a smoother profile of the thermogravimetric curves. As a result, CaCO_3_-filled composites exhibit a more uniform degradation process and an increased residual mass compared to the unfilled polymer.

For the quantitative evaluation of the thermal stability of the polymer systems, the activation energy (E_a_) of degradation was calculated using the isoconversional principle. This approach involves the use of thermogravimetric curves obtained under different heating programs. For this purpose, the TG curves presented above were obtained at three different heating rates, and the E_a_ values were calculated based on them.

**Figure 12:**
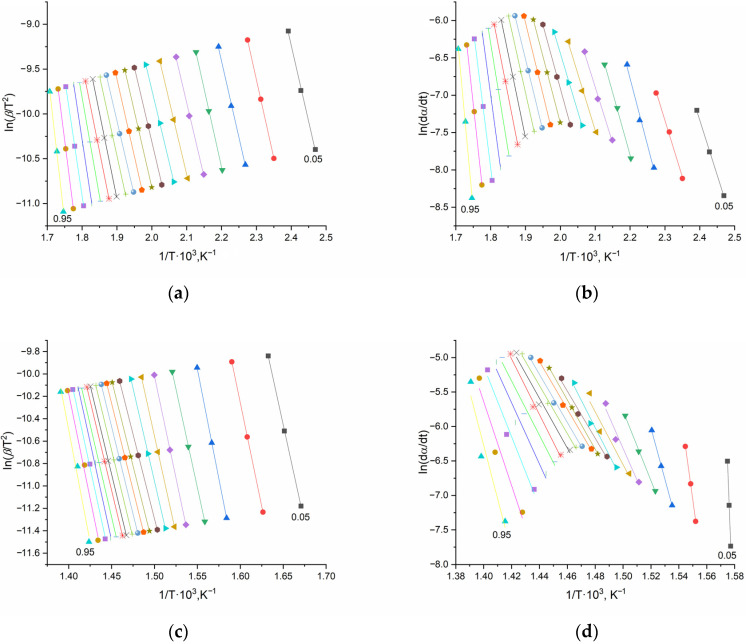
Graphical dependencies of the Kissinger–Akahira–Sunose (**a**,**c**) and Friedman (**b**,**d**) equations for the pEGM45/30% CaCO_3_ system: (**a**,**b**) stage I, (**c**,**d**) stage II. Different colors and symbols correspond to various degrees of conversion (*α* = 0.05–0.95).

As an example, Figure 12 shows the three-point linearization plots for the most thermally stable system, pEGM45/30% CaCO_3_.

As can be seen from the presented plots, the lines constructed using the differential Friedman method exhibit greater scatter of the data points and less pronounced linearity compared to the results obtained by the integral method.

**Figure 13:**
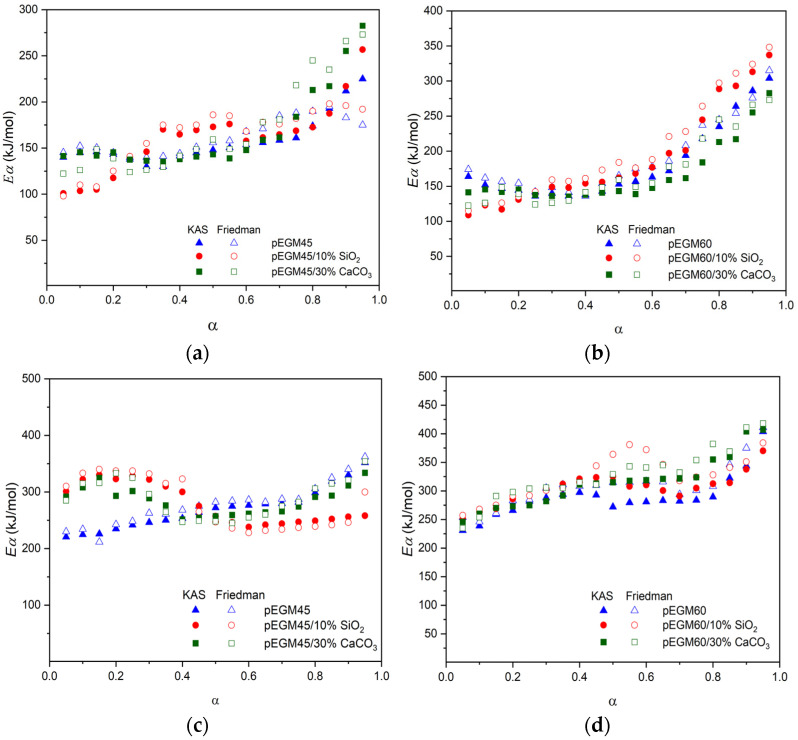
Dependence of activation energy (Ea) on the degree of conversion (*α*) for the pEGM45 and pEGM60 systems: (**a**,**b**) stage I, (**c**,**d**) stage II.

Figure 13 shows the E_a_ values for each individual degree of conversion (*α*) in the degradation range of 0.05–0.95.

The average activation energy values corresponding to both stages of thermal degradation, as well as the total values for each studied system, are presented in Table 4.

A comparative analysis of the obtained E_a_ values for the thermal degradation of pEGM45 and pEGM60 systems containing 10% SiO_2_ and 30% CaCO_3_ showed that, at the initial stages, optimal E_a_ values are observed in accordance with the established patterns. At the second stage, E_a_ increases as expected. The total activation energy is highest for systems with the greatest UP content. It was also found that the fillers increase the E_a_ values of the pEGM systems. For example, the *E_KAS_* value is highest for pEGM60 with fillers, reaching 496 kJ/mol (SiO_2_) and 514 kJ/mol (CaCO_3_). It should be noted that the values obtained by the integral and differential methods (Table 4) show good correlation.

## 4. Conclusions

For the first time, a study was conducted on the rheological properties and the kinetics of the isothermal curing reaction of pEGM systems and their compositions in the presence of the BPO/DMA redox initiating system. It was established that the composition of the studied systems affects their rheological characteristics and the kinetics of low-temperature curing. It has been established that the introduction of mineral fillers alters the flow behavior of pEGM systems, resulting in a transition from Newtonian to pseudoplastic behavior. Three-interval thixotropy tests showed that structural recovery increases with increasing filler content. For the first time, the curing kinetics of pEGM-based systems were studied using DSC. The isothermal curing kinetics of the investigated systems are described by the modified Kamal autocatalytic model, taking into account the diffusion factor. As the UP content increases, the critical degree of curing (*α_c_*) corresponding to the transition from chemical to diffusion control decreases. It was determined that for the pEGM45 system α_c_ is 0.77, while for pEGM60 *αc* is approximately 0.60. It was demonstrated that the application of the modified Kamal autocatalytic model provides an adequate description of the curing kinetics of the studied systems under both moderate and pronounced diffusion limitations. Thermogravimetric analysis revealed that an increase in pEGM content and the introduction of fillers enhance the thermal stability of pEGM45 and pEGM60 systems. The addition of SiO_2_ and CaCO_3_ decreases the maximum decomposition rate (dTGₘₐₓ) and nearly doubles the residual mass compared to the unfilled matrix. The activation energy (E_a_) values for thermal degradation of the pEGM systems, calculated using the Kissinger–Akahira–Sunose and Friedman methods, showed that the introduction of fillers increases E_a_. The *E_KAS_* value was found to be highest for pEGM60 with fillers, reaching 496 kJ/mol (SiO_2_) and 514 kJ/mol (CaCO_3_). The obtained data make it possible to optimize the composition of the systems for low-temperature curing and confirm their potential for use in composite, construction, and coating materials, as well as in adhesive and impregnating formulations.

## Figures and Tables

**Table 1:** Reaction heats obtained from isothermal and dynamic DSC curves.

Temperature, °C	Sample	-∆*H_i_*, J/g	-∆*H_r_*, J/g	-∆*H_tot_*, J/g	*α*
20	pEGM45	255.20 ± 5.59	48.61 ± 1.09	303.81 ± 6.38	0.84
	pEGM45/10% SiO_2_	226.73 ± 4.80	46.44 ± 0.98	273.17 ± 6.28	0.83
	pEGM45/30% CaCO_3_	229.77 ± 5.16	50.44 ± 1.05	280.21 ± 6.16	0.82
20	pEGM60	196.76 ± 4.12	96.92 ± 2.15	293.68 ± 6.16	0.67
	pEGM60/10% SiO_2_	175.95 ± 3.91	90.64 ± 1.87	266.59 ± 5.60	0.66
	pEGM60/30% CaCO_3_	176.94 ± 3.82	95.27 ± 1.99	272.21 ± 5.99	0.65

**Table 2:** Kinetic parameters of the isothermal curing of pEGM-systems at 20 °C.

Sample	Δ*H*_*i*_, (J/g)	Δ*H*_*r*_, (J/g)	Δ*H*_tot_, (J/g)	*α*_max_	*k_1_* ·10^−6^, (s^−1^)	*k_2_* ·10^−4^, (s^−1^)	*m*	*n*	*m* + *n*	*C*	*α_c_*	*R* ^2^
pEGM45	255.20	48.61	303.81	0.84	2.23	7.94	0.543	0.865	1.408	19.7	0.77	0.998
pEGM45/10% SiO_2_	226.73	46.44	273.17	0.83	1.56	5.32	0.511	0.825	1.336	18.5	0.71	0.998
pEGM45/30% CaCO_3_	229.77	50.44	280.21	0.82	1.86	6.65	0.477	0.798	1.275	17.3	0.69	0.997
pEGM60	196.76	96.92	293.68	0.67	0.932	4.56	0.471	0.678	1.149	16.2	0.60	0.999
pEGM60/10% SiO_2_	175.95	90.64	266.59	0.66	0.655	2.26	0.430	0.643	1.073	15.7	0.59	0.995
pEGM60/30% CaCO_3_	176.94	95.27	272.21	0.65	0.781	3.74	0.403	0.599	1.002	14.8	0.56	0.999

**Table 3:** Thermogravimetric data for pEGM systems with fillers.

Sample, mol,%		Temperature Range, °C		dTGmax %/min	Residue, %
p-EGM	Fillers	Stage I	Stage II		
45	-	90~350	350~475	16.7	15.7
	SiO_2_ 10%	95~365	365~480	14.5	21.2
	CaCO_3_ 30%	105~360	360~535	12.9	32.3
60	-	130~380	380~515	16.4	16.5
	SiO_2_ 10%	135~390	390~525	14.7	23.2
	CaCO_3_ 30%	140~390	390~560	13.4	33.6

**Table 4:** Activation energies of thermal degradation for pEGM systems with SiO_2_ and CaCO_3_ fillers.

pEGM45	E_a_, kJ/mol			pEGM60	E_a_, kJ/mol		
	Stage I	Stage II	Σ		Stage I	Stage II	Σ
Kissinger–Akahira–Sunose method							
-	156 ± 1.8	263 ± 4.1	419 ± 5.9	-	181 ± 2.5	289 ± 4.3	470 ± 6.8
SiO_2_ 10%	162 ± 1.2	274 ± 5.3	436 ± 6.5	SiO_2_ 10%	191 ± 2.8	305 ± 5.1	496 ± 7.9
CaCO_3_ 30%	166 ± 1.4	284 ± 2.7	450 ± 4.1	CaCO_3_ 30%	202 ± 3.2	312 ± 5.3	514± 8.5
Friedman method							
-	163 ± 3.6	277 ± 4.8	440 ± 8.4	-	190 ± 4.1	308 ± 8.7	499 ± 12.8
SiO_2_ 10%	165 ± 3.2	291 ± 6.2	456 ± 9.4	SiO_2_ 10%	199 ± 4.4	324 ± 9.1	523 ± 13.5
CaCO_3_ 30%	169 ± 2.7	293 ± 5.4	562 ± 8.1	CaCO_3_ 30%	216 ± 5.6	328 ± 8.4	544 ± 14.0

## Data Availability

The original contributions presented in this study are included in the article. Further inquiries can be directed to the corresponding author.

## Abbreviations

The following abbreviations are used in this manuscript:

AA	acrylic acid
BPO	benzoyl peroxide
DSC	Differential scanning calorimetry
DMA	N,N-Dimethylaniline
FTIR	Fourier Transform Infrared Spectroscopy
IR	Infrared spectroscopy
NMR	Nuclear magnetic resonance
pEGM	Polyethylene glycol maleate
TGA	Thermogravimetric analysis
